# Electrophoretic Deposition of Graphene Oxide on Stainless Steel Substrate

**DOI:** 10.3390/nano11071779

**Published:** 2021-07-08

**Authors:** Dominika Marcin Behunová, George Gallios, Vladimír Girman, Hristo Kolev, Mária Kaňuchová, Silvia Dolinská, Miroslava Václavíková

**Affiliations:** 1 Institute of Geotechnics, Slovak Academy of Sciences, 45 Watsonova Str., 04001 Kosice, Slovakia; sdolinska@saske.sk; 2 Laboratory of General & Inorganic Chemical Technology, School of Chemistry, Aristotle University of Thessaloniki, 54124 Thessaloniki, Greece; gallios@chem.auth.gr; 3 Faculty of Science, Institute of Physics, P.J. Safarik University in Kosice, Park Angelinum 9, 04154 Kosice, Slovakia; vladimir.girman@upjs.sk; 4 Institute of Catalysis, Bulgarian Academy of Sciences, Acad. G. Bonchev St., Bldg. 11, 1113 Sofia, Bulgaria; hgkolev@gmail.com; 5 Faculty of Mining, Ecology, Process Control and Geotechnologies, Technical University of Kosice, 9 Letna Str., 04200 Kosice, Slovakia; maria.kanuchova@tuke.sk

**Keywords:** graphene oxide, stainless steel, electrophoretic deposition

## Abstract

We demonstrated the deposition of the architecture of graphene oxide on stainless steel substrate and its potential environmental application. The synthesis and characterization of graphene oxide were described. The controlled formation of graphene oxide coatings in the form of the homogenous structure on stainless steel is demonstrated by scanning electron microscopy (SEM). The structure, morphology and properties of the material were assessed by Fourier transform infrared (FTIR) spectroscopy, X-ray photoelectron spectroscopy (XPS), Raman spectroscopy, transmission electron microscopy (TEM) and atomic force microscopy (AFM). The morphology and stability of these structures are shown to be particularly related to the pre-treatment of stainless steel substrate before the electrophoretic deposition. This approach opens up a new route to the facile fabrication of advanced electrode coatings with potential use in environmental applications.

## 1. Introduction

Graphene was first observed in 1962 by electron microscope and then rediscovered in 2004 by scientists who were awarded the Nobel Prize in Physics in 2010 [1,2]. It has been studied as a strong nominee for use in many applications including environmental remediation [3,4].

Graphene is a sheet of a single atomic layer of sp^2^ carbon atoms, tightly bound in hexagonal honeycomb lattice [4]. Graphene’s flat honeycomb pattern gives it many unique characteristics and excellent properties such as large surface area, tuneable optical, remarkable thermal and electrical properties and accordingly, mechanical stability [5,6,7,8,9]. It is the first 2D crystal and one of the lightest, strongest, most conductive, transparent and bondable materials known to exist [1]. Graphene oxide (GO) is the functionalized graphene containing oxygen chemical groups including hydroxyl and epoxide groups in the planes along with carboxyl and carbonyl groups at the edges [10]. It is also less expensive than graphene and easier to produce.

The advantage of graphene is based on the amphiphilic character which allows for removing organic and/or inorganic molecules at the nanoscale dimension. Hence, heavy metal ions (Cd^2+^, Pd^2+^, Hg^2+^, Cr^6+^, As^3+^, etc.), synthetic and/or natural organic molecules (dyes), pharmaceuticals (antibiotics), agriculture molecules (pesticides), biomolecules (proteins, DNA, etc.) and mixtures (oil/petrol) are possibly removed by GO [11,12,13].

Nowadays, graphene as sheets (multiple layers), as nanosheets or nanoplatelets and functionalized graphene can be prepared by different methods by micromechanical and electrochemical exfoliation [3], epitaxial growth, chemical vapor deposition (CVD) [14,15] and different chemical methods [16]. Recently, the chemical process with the use of strong oxidizing agents has become a promising approach to produce hydrophilic carbon material graphene. These methods are uncomplicated, low-cost and suitable for large nanoscale graphene/GO production [14]. The process includes significant steps like graphite oxidation, exfoliation of GO and reduction of graphene oxide sheets [14,16,17,18]. Well-known are the Brodie-Staudenmaier-Hofmann and Hummers-Offeman methods and also their modified and improved forms [15]. In these particular methods, originally, graphite powder is chemically reacted with acids (HCl, H_2_SO_4_ and HNO_3,_ etc.) pursued by the intercalation of alkali metal compounds (KClO_3_, KMnO_4_, NaNO_3,_ etc.) into the graphitic layers [19]. In 1859, Brodie determined the synthesis of GO by adding potassium chlorate to the solution of graphite in fuming nitric acid [20]. In 1898, Staudenmaier enhanced this method by using concentrated sulphuric acid in fuming nitric acid and adding a portion of chlorate [21]. In 1928, Hofmann used a mix with concentrated sulphuric acid and potassium chlorate. In 1958, Hummers described the method most commonly used today: the graphite is oxidized by treatment with potassium permanganate and sodium nitrate in concentrated sulphuric acid [8,9,22]. An improved version of Hummers’ method was proposed by Tour´s group (Tour method) at Rice University in 2010 [16]. They have replaced the NaNO_3_ with phosphoric acid in a mixture of H_2_SO_4_/H_3_PO_4_ (9:1) and increased the amount of KMnO_4_. The advantage of this method consists of a greater amount of hydrophilic oxidized GO and the elimination of toxic gases such as NO_2_, N_2_O_4_ or ClO_2_. Hence, this GO is more oxidized and soluble [11,16]. The GO prepared from graphite powder/flake can be easily dispersed in water and has been used for preparing graphitic films. The hydrophilic character of GO allows it to be deposited onto substrates in the form of thin films which is necessary for applications in the electronic industry.

Due to the unique properties of graphene, we demonstrated research based on stainless steel electrode coating using electrophoretic deposition (EPD). EPD is a colloidal two-step process in which charged particles in suspension move towards the electrode of opposite charge. Due to the influence of the electric field, the deposit forms a compact film at the electrode surface [8,9,23]. EPD was discovered in 1808 by Reuss [22,24]. Eventually, EPDs were expanded from being approaches for ceramics and became an important tool in the preparation of advanced materials such as alloys, metals, polymer, oxides or carbides [25,26]. EPD has plenty of advantages such as constant and controllable thickness, smooth, compact surface, etc. [25,27]. Moreover, deposits are produced at room temperature without the production of toxic chemicals. The formation of a homogeneous, closely packed deposit requires the use of a stable suspension in which the particles are dispersed with minimal aggregation. Stable suspensions of a wide assortment of materials can be prepared by tuning the particle-particle interactions, the foremost of which are attractive van der Waals forces and repulsive electrostatic forces. EPD can deposit multiple layers of colloidal graphene and may improve the conductivity of previously oxidized graphene through reduction electrochemistry [8,9,28]. The multi-layered graphene film can be continuously grown through stainless steel (SS) and significantly increase its corrosion resistance [22,29]. A number of researchers have confirmed that steel microstructure was also one of the main factors influencing corrosion resistance [30,31,32,33,34,35,36,37]. The graphene layering on SS also maintains good conductivity and active surface area for adsorbing charged particles [29]. EPD has been shown to be an effective technique for producing graphene layers in liquid suspensions. The aim is to create graphene-based materials including graphene layers and graphene composites. EPD can be applicable for nanocomposite coatings as a promising future for electronics, sensors, energy storage devices, biomedical, energy harvesting and environmental applications [25]. In our research, austenitic stainless steels (SS) were used as platforms to grow graphene sheets and nano-pillars. The stability and response of the metal to the deposition conditions were investigated. Austenitic SS 18/8 alloys are composed of 2 wt% Mo, 18 wt% of Cr and 8 wt% of Ni with the balance Fe and with the alloying elements added to enhance the material resistance to corrosion (Table 1) [38,39]. Growth conditions, including deposition time, voltage, constant current density and growth temperature were investigated. It was shown that the graphene/GO architecture could finely grow on the SS substrate from a few layers thick to complex interconnected macroislands. The appearance of the continuous graphene/GO mesostructure around the stainless steel fibers that form the substrate increases the resistance to corrosion. The intention of this study is to present the EPD technique for the multi-layered architecture of graphene-based materials. GO was coated on the stainless steel substrate. The GO architectures were measured by scanning electron microscopy. The GO morphologies were investigated by scanning electron microscopy (SEM), transmission electron microscopy (TEM) and atomic force microscopy (AFM). Chemical species and functional groups were examined by X-ray photoelectron spectroscopy (XPS), Fourier-transform infrared spectroscopy (FTIR) and Raman spectroscopy.

## 2. Materials and Methods

All chemicals and materials used in this work were used as received.

### 2.1. Preparation of Graphene Oxide

GO was synthesized from fine graphite powder by using the modified Hummers method [8,9,22]. First, graphite powder (0.5 g, Fisher Chemical, G/0900/60, CAS number 7782-42-5, Hampton, New Hampshire, USA) and sodium nitrate (0.5 mL, ITES, Vranov s.r.o., SVK) were stirred in sulphuric acid (23 mL, Mikrochem trade s.r.o., Pezinok, SVK) and cooled to 0 °C in an ice bath for four hours. After 4 h of stirring and powerful agitation, the potassium permanganate (3.0 g, Mikrochem trade s.r.o., Pezinok SVK) was added slowly while the temperature of the suspension was kept near 2 °C. After 1 h of stirring, the ice bath was removed and the mixture was heated at 35 °C for another hour. Then, deionized water (46 mL) was added and the solution was stirred for 2 h at 90–95 °C. The reaction mixture was cooled at room temperature. Additional deionized water (100 mL) was added, the solution was stirred for another 1 h, followed by slow addition of 30% hydrogen peroxide (10 mL). After 1 h of stirring, GO was formed, turning the color of the solution from yellow to orange and finally to dark brown. The mixture was filtered and rinsed with 5% HCl aqueous solution followed by washing with distilled water to remove the acid. The oxidation product was washed, centrifuged (ROTINA 380, Hettich Zentrigfugen 4000 rpm/40 min) several times until the pH reached 6 and then filtered, then dried in a vacuum oven and examined by XPS, TEM, SEM, AFM and FTIR techniques.

#### Modified Tour Method

For the Tour method [16], a 9:1 mixture of concentrated H_2_SO_4_/H_3_PO_4_ (360:40 mL) was added to a mixture of graphite powder (3.0 g, Fisher Chemical, G/0900/60, CAS number 7782-42-5, Hampton, New Hampshire, USA) and KMnO_4_ (18.0 g, Mikrochem trade s.r.o., Pezinok, SVK). The reaction was heated to 50 °C and stirred for 12 h continuously. The reaction was cooled to room temperature and poured onto ice cubes (~400 mL) with 30% H_2_O_2_ (3 mL). The mixture was not sifted through a metal standard testing sieve (300 μm) because we used fine graphite powder instead of graphite flakes. The filtrate was centrifuged (ROTINA 380, Hettich Zentrigfugen 4000 rpm/4 h) and the supernatant was decanted away. The remaining solid graphene material was then purified to remove undesired elements in sequence with 200 mL of water, 200 mL of 30% HCl and 200 mL of ethanol for each wash. The filtrate was stirred, centrifuged (4000 rpm/4 h) and the supernatant was decanted away. The material remaining after this extended, multiple-wash process was coagulated with 200 mL of diethyl ether (ITES, Vranov s.r.o., SVK), and the resulting suspension was filtered over a PTFE membrane with a 0.45 μm pore size. The solid obtained on the PTFE filter was vacuum-dried overnight at room temperature [16]. GO prepared from powder graphite can be dispersed in water and has been used on a large scale for preparing large thin films [16].

### 2.2. Deposition of Reduced Graphene Oxide on Stainless Steel Substrate

**Figure 1:**
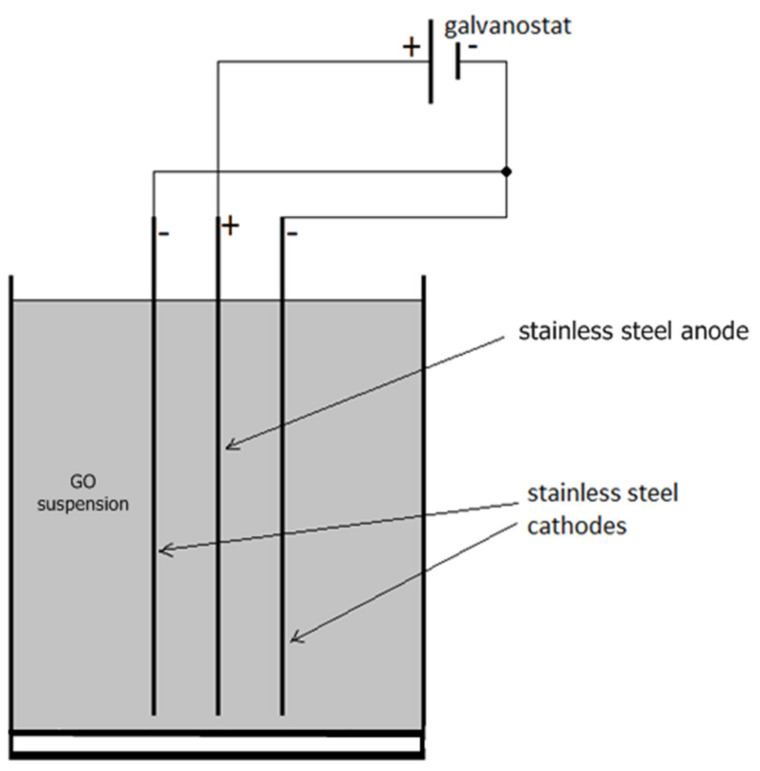
The scheme of typical EPD for deposition of GO on the SS substrate with one positive and two negative electrodes aligned in parallel.

GO architecture was easily and effectively synthetized by electrophoretic deposition. In this process, 150 mg of GO powder was dispersed in 100 mL deionized water and sonicated an ultrasonic homogenizer (KRAINTEK, Podhájska, SVK) for 2 h at room temperature. SS substrate (7.4 × 4.5 cm) and acrylic glass cell were well cleaned and polished. The special pre-treatment method was used on the surface of the SS electrode. Two cleaned SS plates served as cathodes, one piece of SS in the middle served as the anode (Figure 1) and electrodes were connected to the power supply (Statron 2225, Bad Herrenalb, Germany). Other materials such as aluminum foil, copper or nickel substrate could also be used as anode material [30]. The direct current (DC) voltage of 10 V was applied between the SS substrate for 8 min during the deposition. Negatively charged GO migrated towards the positive electrode (anode) due to the influence of an electric field. Under the proper condition, particles aggregate to form the layered film that adheres to the surface of SS. After deposition, the samples were air-dried for 24 h. The process was repeated 3 times; homogenous multilayers of closely packed deposits were created. The anode was then baked for 24 h at 80 °C initially and then at 300 °C for 2 h to partially reduce the GO and improve conductivity [40]. 

### 2.3. Characterization

The obtained GO substrates were first characterized by the scanning electron microscope (SEM) (MIRA 3 FE-SEM microscope TESCAN, Brno, Czech Republic) equipped with a high-resolution cathode (Schottky field emitter) and with three-lens Wide Field Optics^TM^ design and Energy-dispersive X-ray detector (EDX) (Oxford Instruments, Abingdon, UK).

The morphology and structure were analyzed using the transmission electron microscope (TEM) (model JEOL JEM 2100F UHR). The experiments were conducted at 80 kV to 200 kV accelerating voltage with ultrahigh resolution (0.19 nm) and maximum magnification of 1.5 Mx. The microscope is equipped with a Schottky FEG gun that produces high brightness, and the three-stage condenser with two-stage objective, three-stage intermediate and projector lenses. The electron diffraction techniques such as selected area diffraction, nano-beam diffraction and convergent beam diffraction were used for structural or crystallographic analysis in TEM mode.

X-ray photoelectron spectroscopy (XPS) measurements have been carried out on the SPECS (SPECS GmbH, Berlin, Germany) electron spectrometer equipped with PHOIBOS 100 SCD analyzer. Base pressure in the analysis chamber was 5 × 10^−10^ mbar (2 × 10^−8^ mbar during the measurements). The measurements have been performed using the twin anode MgKα/AlKα non-monochromated X-ray source, used with excitation energies of 1253.6 and 1486.6 eV, respectively. The data were evaluated by SpecsLab2 CasaXPS software (Casa Software Ltd.). The energy scale has been calibrated by normalizing the C1s line of hydrocarbons to 285.0 eV. The processing of the measured spectra included subtraction of X-ray satellites and Shirley-type background [41]. The peak location and areas were evaluated by a symmetrical Gaussian-Lorentzian curve fitting. The relative concentrations of the different chemical species were determined after normalization of the peak areas to their photoionization cross-sections, calculated by Scofield [42].

The chemical species were examined by Fourier-transform infrared spectroscopy using the Bruker Tensor 27 FTIR spectrometer equipped with a dGTS/KBr detector. The infrared spectra were obtained using the KBr disc technique in the abs mode (64 scans, 4000–400 cm^−1^ spectral range and resolution of 4 cm^−1^). 

The functionalization of graphene oxide was studied by Raman spectroscopy using the microscope XploRA ONE Horiba Scientific Jobin Yvon with a laser wavelength of 532 nm and magnification objective of 20x in range of 100 to 4000 cm^−1^. The laser power used for the measurements was limited by the filter to 10% to a maximum of 100 mW to decrease the probability of sample damage/oxidation. The spectra were collected from three or four different spots on the sample. The exposure time was 10 s and two scans were collected. 

The topology measurements were performed by atomic force microscopy Dimension ICON by Veeco/Bruker microscope in the ScanAsyst scanning mode, which allowed for recording high definition surface topology data in height and Peak Force Error mode. The last mode provides increased sharpness of the edges of the surface features, when commonly used height mode image is blurry or distorted by some artefacts. Image processing was performed in NanoScope Analysis (software distributed by Bruker with AFM machines) and Gwyddion—a modular, multiplatform, open-source software for scanning probe microscopy data processing [30].

## 3. Results and Discussion

### 3.1. Characterization of Graphene Oxide

The GO prepared by Tour method (TGO) provides the greater amount of hydrophilic oxidized graphite material than GO by Hummers method (HGO). More advantages of the Tour method are the simplicity of the process, higher yield and elimination of toxic gases during preparation.

**Figure 2:**
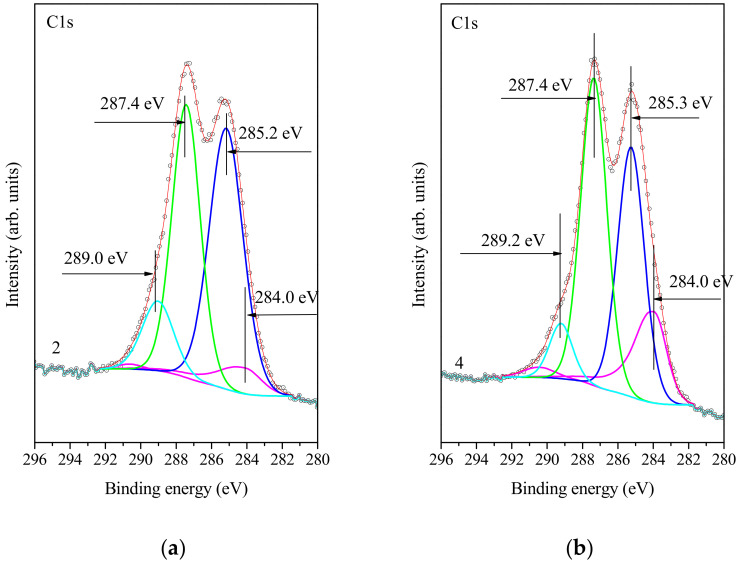
(**a**) XPS measurement of TGO, (284.0 eV C=C; 284.4 eV C-O; 289.0 eV C=O; (**b**) XPS of HGO, (284.0 eV C=C; 285.3 eV C-O; 287.4 eV C=O and 289.2 eV O-C=O).

The oxygen speciation was studied by X-ray photoelectron spectroscopy (XPS). Figure 2 shows the C1s core-level XPS spectra of graphite and graphene oxides. The spectra of GO show the peak at 284 eV, attributed to carbon with sp^2^ and sp^3^ hybridization. Furthermore, it is obvious that TGO is more oxidized than the HGO. The C/O ratio was not used due to the difficulty of fully dehydrating the GO samples. The C1s spectra of TGO were analyzed by SpecsLab2 CasaXPS software (Casa Software Ltd.) with each spectrum in four peaks that correspond to the following functional groups: sp^2^ (C=C, 284.0 eV), epoxy/hydroxyls (C-O, 285.2 eV), carbonyl (C=O, 287.4 eV) and carboxylates (O-C=O, 289.0 eV).

The C1s spectra of HGO correspond to sp^2^ (C=C, 284.0 eV), epoxy/hydroxyl (C-O, 285.3 eV), carbonyl (C=O, 287.4 eV) and carboxylates (O-C=O, 289.2 eV).

**Figure 3:**
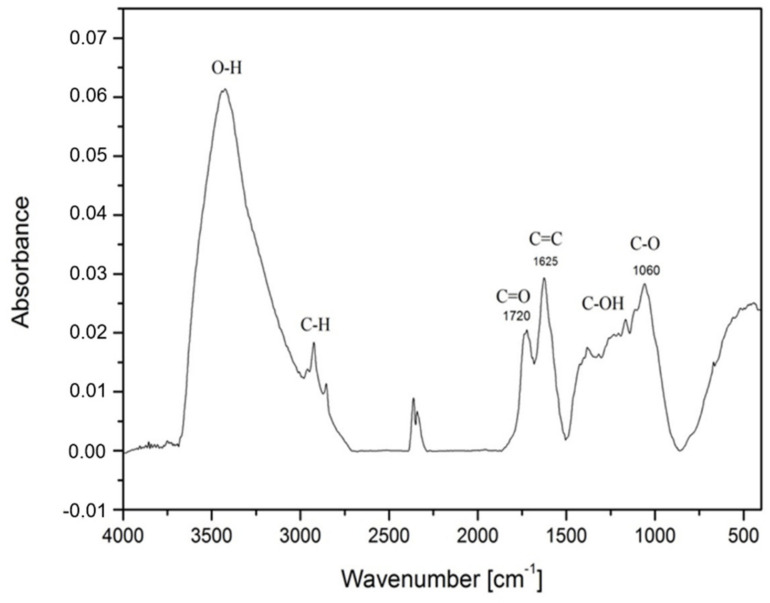
FTIR spectrum of GO prepared by Tour method.

FTIR spectra of TGO (Figure 3) were recorded and the following functional groups were identified and confirmed in the sample. IR spectrum shows a strong peak at 1720 cm^−1^ which corresponded to stretching vibrations of the carbonyl group C=O. The highest peak, 3475 cm^−1,^ was caused by the hydroxyl OH group and defined the stretching vibrations C-OH and water. The peak at 1625 cm^−1^ represented C=C from unoxidized sp^2^ C-C bonds. Stretching vibrations of 1060 cm^−1^ characterized the epoxy functional group C-O whereby a peak at 1210 cm^−1^ was usually attached to a stretching vibration of C-OH. 

**Figure 4:**
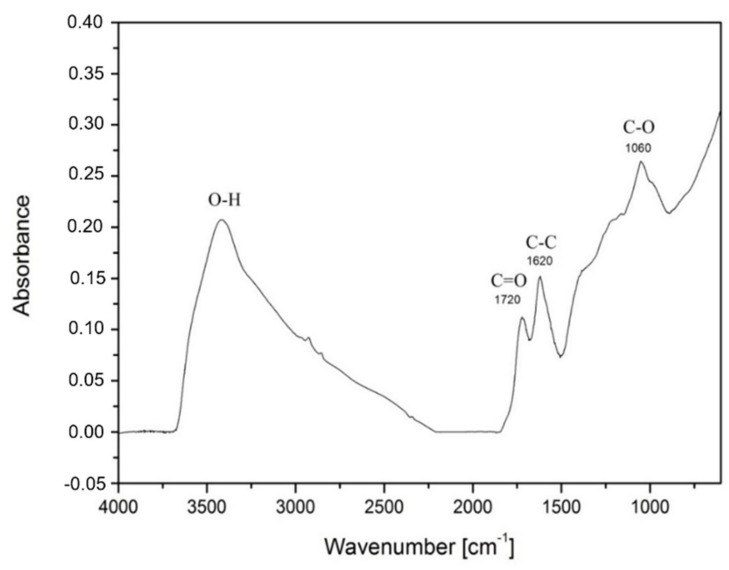
FTIR spectrum of GO prepared by Hummers method.

FTIR of HGO confirmed the oxygen functional groups. A section of the IR spectrum confirmed the carbonyl functional group C=O at 1720 cm^−1^, C=C 1620 cm^−1^ and epoxy functional group C-O 1060 cm^−1^. The script on Figure 4 deviated considering the high water content or O-H and H-O-H groups, which presented a peak at 3500–3300 cm^−1^. The sample was difficult to fully dehydrate. Each synthesis created the distinguished GO which is why the results are slightly different.

Based on the literature, the FTIR signals are initiated to hydroxyl group (OH) 3400 cm^−1^, epoxy (C-O) 1090 cm^−1^ and ketone (C=O) 1700 cm^−1^ [11]. Another group of scientists obtained the following vibration frequencies: hydroxyl group 3050–3800 cm^−1^, carbonyl 1750–1850 cm^−1^, carboxyl 1650–1750 cm^−1^, C=C 1500–1600 cm^−1^ and ethers/epoxy 1000–1280 cm^−1^ [43,44]. Authors argue that peaks (~1730 cm^−1^ and ~1620 cm^−1^) are not different carbonyl/carboxyl groups but the doublet of keto-enol tautomers [45].

**Figure 5:**
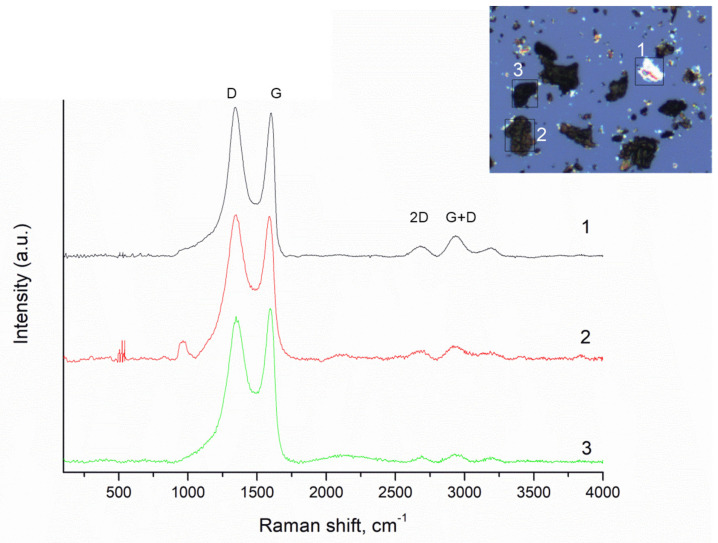
Raman spectrum of GO prepared by Hummers method with proposed deconvolution of the D, G, D′, 2D and S3 bands. GO creates two main peaks: G (~1602 cm^−1^), a primary in-plane vibrational mode, and 2D (2700 cm^−1^), a second-order overtone of a different in-plane vibration, D (1350 cm^−1^). D and 2D peak positions are dispersive (dependent on the laser excitation energy).

Raman spectroscopy gave information about the structural characteristics of HGO and TGO. The D (~1350 cm^−1^) peak position is attributed to a structural disorder like defects and/or edges in the material. The D peak corresponds to the breathing mode of κ-point phonons of A1g symmetry, while the G (~1602 cm^−1^) peak corresponds to the E2g phonon from the stretching of carbon sp^2^ atoms [46] (Figure 5). The Raman spectrum is also modified due to the disorder in the sp^2^ structure after oxidation of graphite powder and because of the attachment of hydroxyl and epoxide groups on the carbon structure. The stronger the D band over the G band depends on the functionalization degree of GO. The intensity ratio between these two peaks indicates the quality of the material. The 2D band (2700 cm^−1^) is known as the indicator of the number of graphene layers. Prepared HGO contains mostly a few layers with some defects. If you compare it with the D and G band, the peak is very small, but it may be improved by reducing the number of GO layers. For instance, there is a bold difference between curve number three (thick layer) and number one (thin layer). The S3 peak (~2921 cm^−1^) is a second-order peak derived from the D-G peak combination. The S3 band is observed, which confirms the presence of a disordered structure for GO. The differences among the positions of the individual bands are minimal.

**Figure 6:**
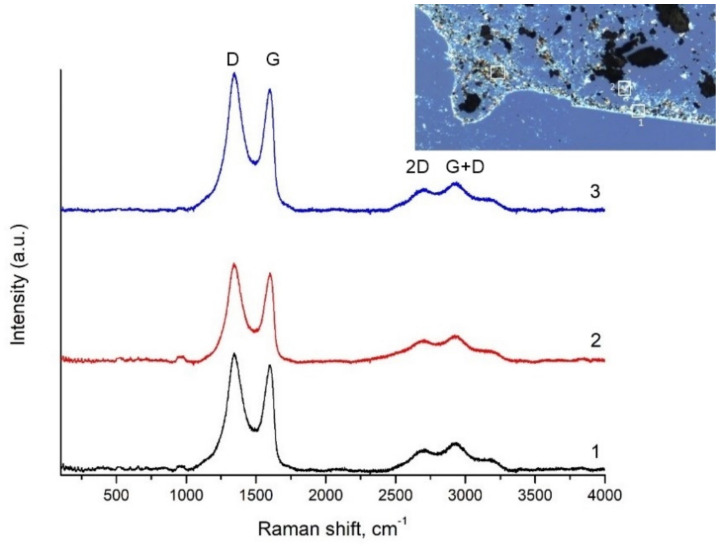
Raman spectrum of GO prepared by Tour method with proposed deconvolution of the D, G, D′, 2D and S3 bands. GO creates two main peaks: G (~1605 cm^−1^), a primary in-plane vibrational mode, and 2D (2700 cm^−1^), a second-order overtone of a different in-plane vibration, D (1350 cm^−1^). D and 2D peak positions are dispersive (dependent on the laser excitation energy).

Raman spectra of TGO as seen in Figure 6 show strong D (~1350 cm^−1^) peaks and G (~1605 cm^−1^) peaks, suggesting very small crystal sizes. Because the D band is stronger than the G band, the functionalization degree of TGO is higher, which was confirmed also by the XPS method. In general, GO has a highly disordered structure due to many functional groups in the architecture formed during oxidation of graphite powder/flakes.

**Figure 7:**
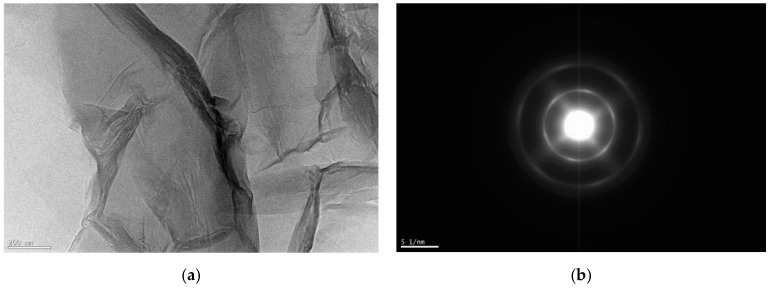
(**a**) TEM of TGO magnification 15 kx, (**b**) electron diffraction micrograph of TGO.

**Figure 8:**
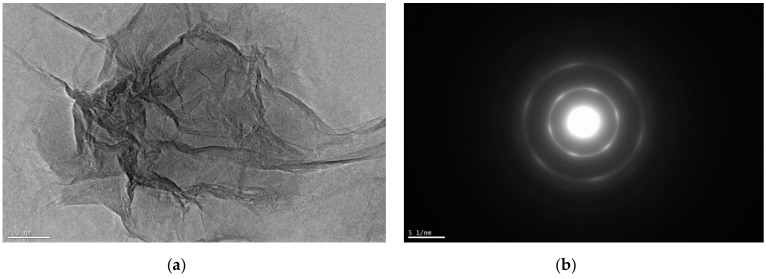
(**a**) TEM of HGO magnification 15 kx, (**b**) electron diffraction micrograph of HGO.

Transmission electron microscopy (TEM) images of two samples support the assertion that TGO has a more regular structure than HGO (Figure 7 and Figure 8). All procedures produce large flakes of GO which are a few layers thick, but the electron diffraction patterns indicate the crystallinity differences. The TGO is highly oxidized; however, it possesses the pointed electron diffraction pattern and more carbon framework. Darker areas indicate the thicker nanostructure of several GO layers. The higher transparency areas indicate the thinner composition of GO layers due to the stacking of exfoliated nanostructure. Delaminated graphene layers are shown by the TGO sample.

**Figure 9:**
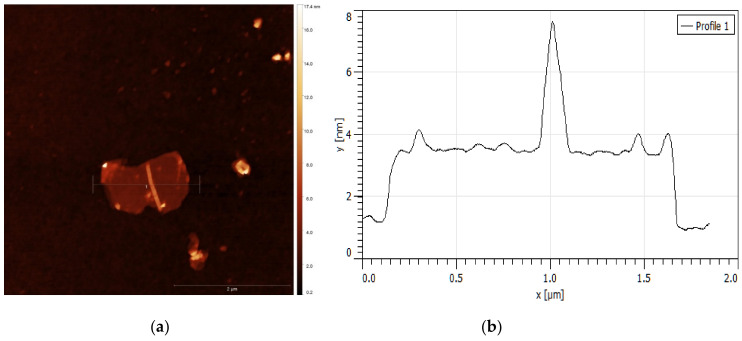
(**a**) AFM images of layered TGO nanosheets on SiO_2_ profile with (**b**) corresponding height profile.

**Figure 10:**
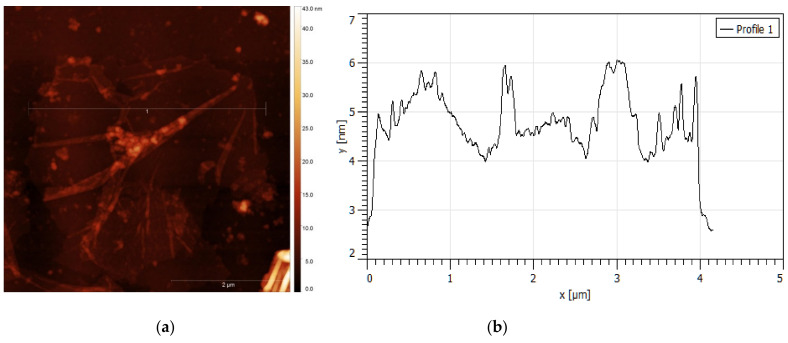
(**a**) AFM images of layered HGO nanosheets on SiO_2_ with (**b**) corresponding height profile.

Atomic force microscopy is one of the main methods for direct identification and confirmation of thickness and roughness of single and layered graphene material. GO was sonicated before detection to avoid agglomerates in the sample. Freshly AFM probe apex and SiO_2_ surface were used to eliminate defects. The GO films are known to present wrinkles and overlaps. TGO and HGO consist of a few stacked layers. Topographic images of GO layers are shown in Figure 9 and Figure 10 together with the height profiles taken along lines. Figure 9 shows an individual TGO flake, which is less than 2 μm in diameter and based on height profile, only 2 nm thick. The thickness of the monolayer GO flake is in the area of 0.7–1 nm; accordingly, the shown flake consists of two layers [16,17]. However, discrepancies in values for single-layer graphene measured by various modes of AFM are in the range of 0.3 to 1.7 nm [47]. Figure 10 shows an HGO flake of 4 μm in diameter which is approximately 3 nm thick and corresponds to three GO layers. HGO has a significantly rougher and wrinkled surface than TGO.

### 3.2. Electrophoretic Deposition

GO was located with interconnection at the surface of stainless steel electrodes by the electrophoretic deposition method. In all experiments, DC voltages were applied between electrodes placed in the GO suspensions at a distance of 1 cm. Negatively charged GO was deposited on the anode, while deposition was not observed on cathodes. In the absence of voltage, no visible film was formed on all electrodes. Moreover, water electrolysis reaction occurs in the electrochemical system and is an essential step to the mechanism of GO deposition. Water decomposed to produce oxygen and dissolved protons at the anode, while electrons from the cathode combined with protons to form hydrogen. These gases were observed as the bubble´s cloud generated on the electrode surfaces [28]. 

**Figure 11:**
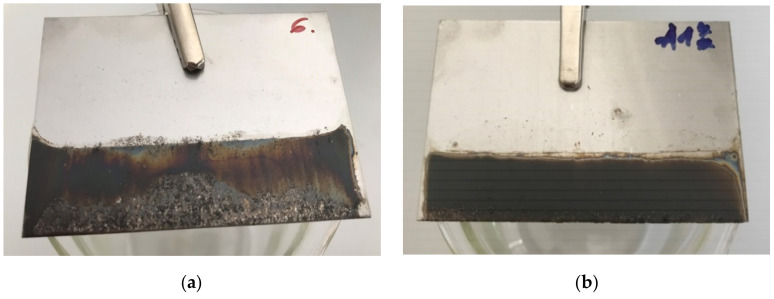
(**a**) First application of GO by EPD, (**b**) third application of GO by EPD.

Figure 11a shows the film deposited onto SS from suspension with a pH of 7 using 10 V for 8 min. With the same deposition conditions for both GO, the thicker, more visible and homogenous layer was obtained with TGO. Hence, from here on only TGO was used and investigated. 

**Figure 12:**
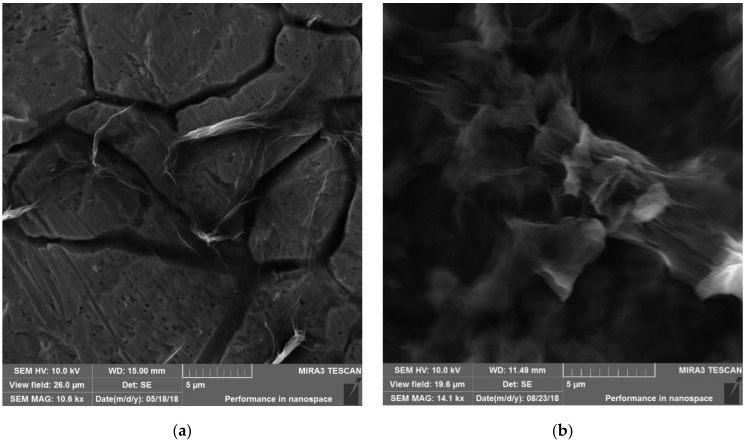
(**a**) First deposition of GO by EPD, magnification 10.6 kx, (**b**) third deposition of GO by EPD, magnification 14.1 kx.

**Figure 13:**
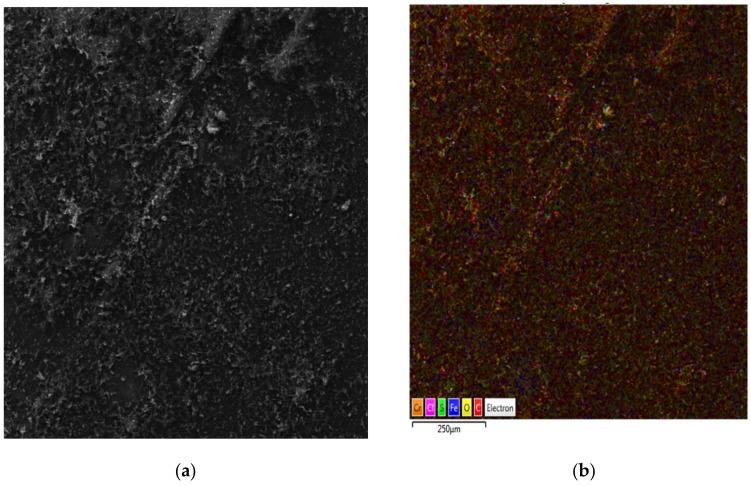
Characterization of electrophoretically deposited GO film on stainless steel (scale 200 μm). Films with cover microstructure are shown in micrographs, (**a**) SEM image and (**b**) EDX image.

The deposition is thicker with the third application of the GO layers as it is shown in Figure 11b. The same conditions were used for all depositions. Obviously, the film deposited on both sides of the electrode that was submerged in the liquid. The outlines of GO sheets were visible (SEM micrographs, Figure 12). The structure of GO was highly wrinkled. The void spaces that were visible with one deposition (Figure 12a) disappeared with three depositions of GO and the thicker and more homogeneous surface was observed (Figure 12b). The results of the scanning electron microscopy (SEM) and energy dispersive X-ray detector (EDX) analyses of the GO deposited on SS with the elemental mapping are given in Figure 13a,b. These results are in good agreement with the results of the TEM analysis.

**Figure 14:**
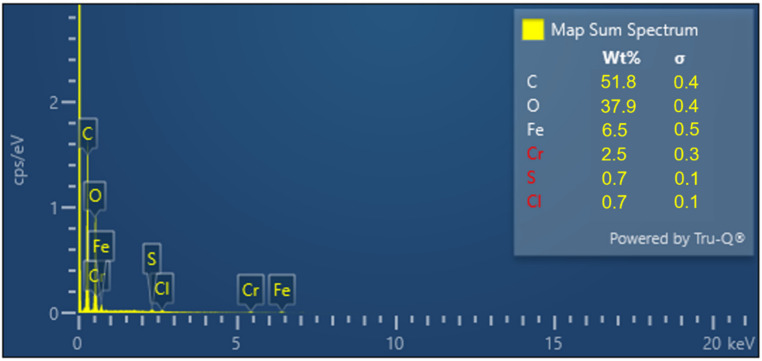
Identification peaks of the elemental composition of GO-based electrode.

The GO morphology and chemical composition of elements in the analyzed structures were also confirmed by the application of other methods of measurement, for example scanning electron microscopy with the EDX detector. Figure 13a shows the homogenous multilayer structure of GO on SS substrate. EDX maps are shown in Figure 13b; carbon and oxygen are detected on the surface of samples and the presence of iron and chromium on the surface is the striking difference due to the layer of GO. The amount of elements detected using various methods is summarized in Figure 14.

**Figure 15:**
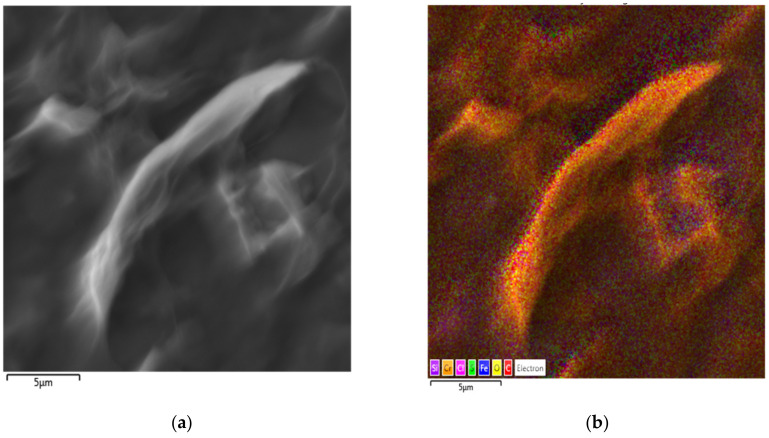
SEM characterizations of electrophoretically deposited GO film (scale 5 μm); (**a**) SEM image, (**b**) EDX image.

**Figure 16:**
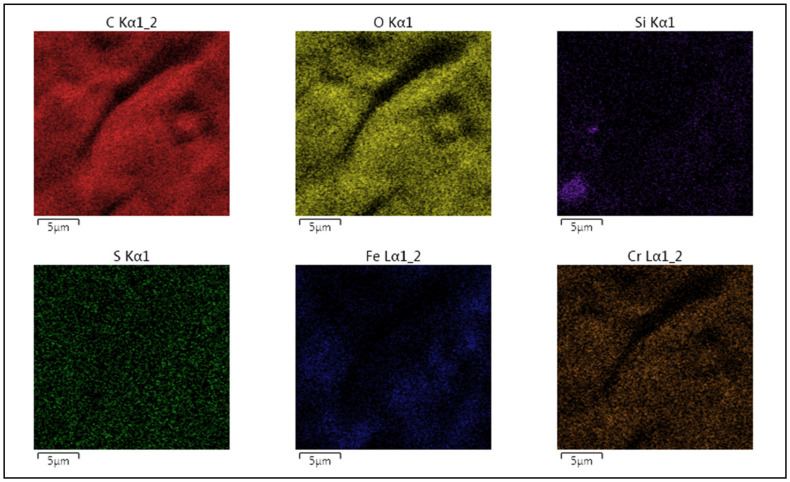
Distribution of C, O, Si, S, Fe and Cr elements obtained by mapping of the GO samples on stainless steel electrodes.

The detailed GO morphology and chemical composition of elements are shown in Figure 15 and Figure 16. The analyzed samples show these homogenous multilayered structures of GO. EDX maps are given in Figure 16 carbon and oxygen are detected on the surface of samples and also iron and chromium were found on the surface with the light pattern due to the layer of GO.

The resulting GO film is quite flexible has a homogenous mesoporous structure with GO macro-islands present on the surface of stainless steel. The GO coatings increased the corrosion resistivity and specific surface area and improved the electrical properties.

## 4. Conclusions

Graphene oxide was successfully produced by oxidizing graphite powder by the modified Hummers’ and Tour methods. The Tour method produced more hydrophilic material that allowed GO to dissolve in solvents, i.e., water. Thus, TGO was preferable, and it allowed simple deposition of multiple layers of GO on austenitic stainless steel 18/8 electrodes from water suspensions by applying DC current (electrophoretic deposition). The thick layer was deposited on stainless steel electrodes; the multilayer GO formation was confirmed by scanning electron microscope. Graphene oxide nanomaterials were grown vertically on stainless steel substrate to form a three-dimensional architecture.

The mechanism of electrophoretic deposition is still not clear and needs to be investigated further. However, the deposition of the GO layers could be effectively controlled by varying important parameters such as contact time, temperature, solution agent, direct current density, direct current voltage, etc. An important step was the preparation of a stable GO suspension which was obtained by combining mixing (3 h in magnetic stirrer) and sonication (2 h). The minimum contact time for homogenous deposition was 8 min with DC current intensity of 1 to 1.5 A. The technique was effective and could be used to produce large-area films of colloidal graphene oxide on stainless steel substrates.

The innovative surface pre-treatment was required for a successful deposition which consists of detergents such as isopropyl alcohol, nitric acid, ultrapure water and sonification. An important step was the preparation of a stable GO suspension which was obtained by combining mixing (3 h in magnetic stirrer) and sonication (2 h). The deposition was repeated three times to create a protected film based on graphene oxide. There was no need to apply organic solvents as the water suspension was used. The minimum contact time for homogenous deposition was 8 min with DC current intensity of 1 to 1.5 A. The anode was then baked for 24 h at 80 °C initially and then at 300 °C for 2 h to partially reduce the GO. Stainless steel anode cannot be used during the electrolysis process because it will release many ions in the electrolyte solution depending on the nature of the solution. Thus, we decided to create a protected film based on graphene oxide. Graphene coatings are claimed to be the thinnest known corrosion-protecting coating. Such a layer of graphene oxide created a protected film. Inclusions or microcrevices were not created in the steel matrix, which could have induced pitting corrosion. The coating of electrodes prepared without surface pre-treatment were not successful and pitting was observed. The initial corrosion induced by inclusions and microstructure should be examined by SEM/EDX technique. Inclusions were not observed. We assume that the pre-treatment of the electrode, stability of the graphene oxide suspension, low DC current density, as well the time reduction could also affect potential corrosion resistivity. The good performance was related to the barrier nature of the GO film and shielding of the local electrical fields hindering the evolution of local processes, decreasing the tendency to metastable pitting.

Recent research shows that the removal of hazardous chemical materials using graphene oxide is a quite novel approach in the treatment of wastewaters. This unique material offers unconventional physico-chemical properties that can significantly improve the removal efficiency while at the same time restrict the negative impact on the environment and human health. Graphene oxide is capable of simultaneously removing a broad class of molecules and displaying a strong affinity for the adsorption of contaminants and convert them to harmless products. In addition, electrophoretic deposition can be an effective method for the preparation of graphene-based nanostructures for various applications.

## Figures and Tables

**Table 1:** Elemental composition of the austenitic stainless steel substrate 18/8.

Elements	Mo	Cr	Ni	Fe
[wt%]	2	18	8	71

## Data Availability

Not applicable.
